# Inflammatory cytokines as potential biomarkers for early diagnosis of severe malaria in children in Ghana

**DOI:** 10.1186/s12936-023-04652-w

**Published:** 2023-07-31

**Authors:** Elizabeth Obeng-Aboagye, Augustina Frimpong, Jones Amo Amponsah, Samuel E. Danso, Ewurama D. A. Owusu, Michael Fokuo Ofori

**Affiliations:** 1 Department of Medical Laboratory Sciences, School of Biomedical and Allied Health Sciences, College of Health Sciences, University of Ghana, Accra, Ghana; 2 Department of Immunology, Noguchi Memorial Institute for Medical Research, College of Health Sciences, University of Ghana, Accra, Ghana; 3 GA East Hospital, Accra, Ghana

**Keywords:** Severe malaria, Uncomplicated malaria, Biomarker, Pro-inflammatory cytokines, Anti-inflammatory cytokines

## Abstract

**Background:**

Severe malaria (SM) is a fatal multi-system disease which accounted for an estimated 619,000 deaths in 2021. Less than 30% of children presenting with SM are diagnosed and treated promptly, resulting in increased mortality and neurologic impairments in survivors. Studies have identified cytokine profiles that differentiate the various clinical manifestations of malaria (severe and uncomplicated). However, the diagnostic capability of these cytokines in differentiating between the disease states in terms of cut-off values has not yet been determined.

**Methods:**

The plasma levels of 22 pro-inflammatory cytokines (Eotaxin/CCL 11, interferon-gamma (IFN-γ), interleukin (IL)- 2, IL-6, IL-1β, IL-12p40/p70, IL-17A, RANTES, MCP-1, IL-15, IL-5, IL-1RA, IL-2R, IFN-α, IP-10, TNF, MIG, MIP-1α, MIP-1β, IL-7, IL-8 and Granulocyte Macrophage-Colony Stimulating Factor (GM-CSF), and 3 anti-inflammatory cytokines-(IL-4, IL-13 and IL-10) in patients with SM, uncomplicated malaria (UM) and other febrile conditions, were measured and compared using the Human Cytokine Magnetic 25-Plex Panel. The receiver operating characteristic (ROC) curve analysis was used to determine the diagnostic value of these cytokines.

**Results:**

The level of the pro-inflammatory cytokine, IL-17A, was significantly higher in the SM group as compared to the UM group. Levels of the anti-inflammatory cytokines however did not differ significantly among the SM and UM groups. Only IL-1β and IL-17A showed good diagnostic potential after ROC curve analysis.

**Conclusion:**

The data show that levels of pro-inflammatory cytokines correlate with malaria disease severity. IL-1β and IL-17A showed good diagnostic potentials and can be considered for use in clinical practice to target treatment.

## Background

Globally, malaria is the most prevalent infectious disease affecting human health [1] and takes an estimated 435,000 lives each year, especially among young African children [2]. The 2022 world malaria report gave an estimation of 247 million cases and 619,000 deaths worldwide in 2021 [3]. Unfortunately, children aged under 5 years, remain the most vulnerable population affected by malaria, and accounts for 67% (274,000) of these deaths.

Generally, malaria is an uncomplicated febrile illness that resolves with time [2]. However, in approximately 1% to 2% of cases, malaria progresses to a severe disease that is lethal. This progression may be influenced by age, exposure and immune status [4], therefore, children under 5 years and the aged population in endemic areas, as well as pregnant women and HIV patients are at risk of severe malaria [5]. Severe malaria (SM) is a complex multi-system disorder [6] caused almost exclusively by *Plasmodium falciparum* [7]. SM is of paramount importance because its mortality rate remains unacceptably high at 58% from the year 2000 to 2019 [8].

Inflammatory cytokines have gained the interest of researchers in recent times as they have been implicated in the pathogenesis of many diseases despite their complex association with multiple regulatory pathways. Unregulated levels and a change in their expression patterns are said to have the potential as biomarkers for several diseases [9]. Several studies have identified and compared cytokine profiles in different disease and their levels are said to correlate with malaria disease severity and death [10–13]. Inflammatory cytokines are categorized based on their effect on inflammation as pro-inflammatory, which include IL-1, IFN-γ, tumour necrosis factor (TNF), IL-12 and IL-18, or anti-inflammatory, which include IL-4, IL-10, IL-13 and transforming growth factor-beta (TGF-β) [10]. Pro-inflammatory cytokines promote inflammation and tissue damage [14], whereas anti-inflammatory cytokines prevent the damaging effects of pro-inflammatory cytokines by resolving inflammation and facilitating tissue repair [14, 15].

Progression of uncomplicated malaria to severe disease is therefore warranted by an imbalance in the levels of pro- and anti-inflammatory cytokines, that is, lower levels of anti-inflammatory cytokines compared to pro-inflammatory cytokines [16, 17]. Several studies have identified cytokine profiles during various clinical manifestations of malaria (SM and UM). For instance, increased levels of IL-12, IL-6, IL-10, IL-1β and IFN-γ have been identified during severe malaria whereas low levels of 1L-12p70, IL-10, IFN-α, and IFN-γ have been associated with SM in children [10, 13, 18, 19]. However, the diagnostic capability of these cytokines in differentiating between the disease states (SM and UM) in terms of cut-off values was not established.

This study aims to measure the levels of inflammatory cytokines among the various groups of participants (SM, UM and Febrile Controls), and compare the levels in each group to that of the SM group, establishing the degree of significance of the differences between the levels. Additionally, the diagnostic potential of these cytokines to differentiate between SM and UM cases was determined, with their corresponding cut-off values, sensitivities and specificities.

## Methods

### Study sites

This was a case–control study conducted at the Princess Marie Louise (PML) Children’s Hospital located in James Town, Accra and the Hohoe Municipal hospital. The PML is a 74-bed facility, which is NHIS accredited and provides specialist care in paediatric radiology, paediatric surgery, dietetics, disease control, and nutrition rehabilitation among others. There is also an outpatient department (OPD) that provides early diagnosis, curative, preventive and rehabilitative care on an ambulatory basis. The hospital, being a secondary institution, receives cases referred from all over the country as well as neighboring countries. The Hohoe Municipal Hospital is a 178-bed capacity hospital located in Hohoe, Volta Region, Ghana. It provides general services in health care and has the highest OPD attendance in the region, which stands for about 112,000 annually. Its location, which is central to the Oti and Volta Regions makes it a major referral point. Archived clinical malaria samples were obtained from children who were permanent residents of Hohoe, an area known for its high malaria transmission intensity, with an entomological inoculation rate of 65 infectious bites per person per year [20]. The febrile controls were obtained from PML.

### Sample collection

A convenient sampling method was used because of the low prevalence (1%) of severe malaria. Whole blood samples were obtained from 57 children; 37 with clinical malaria without sepsis (27 SM, 10 UM) and 20 children with non-malaria related fever (febrile controls) after informed consent was obtained from parents/guardians of participants. Children with SM were classified based on high parasitaemia under the light microscope, and symptoms such as severe anemia, acute respiratory distress, hypoglycaemia and multiple convulsions. Those with UM presented with symptoms such as fever, headache, anorexia, vomiting and abdominal discomfort. Clinical malaria samples were obtained as archived samples collected under the Institutional Review Board of the Noguchi Memorial Institute for Medical Research (Study No. 026/13-14), University of Ghana and the Ghana Health Service Ethical Review Committee (GHS-ERC 08/05/14). The blood samples were centrifuged at a speed of 3000 rpm for 5 min to obtain the plasma. The plasma was pipetted into Eppendorf tubes and stored at −80ºC until time for experiments.

### Determination of inflammatory cytokine levels

A Human Cytokine Magnetic 25-Plex Panel (Thermo Fisher Scientific Corporation, United States of America) was used to estimate the levels of these cytokines; 22 pro-inflammatory cytokines (Eotaxin/CCL 11, interferon-gamma (IFN-γ), interleukin (IL)- 2, IL-6, IL-1β, IL-12p40/p70, IL-17A, RANTES, MCP-1, IL-15, IL-5, IL-1RA, IL-2R, IFN-α, IP-10, TNF, MIG, MIP-1α, MIP-1β, IL-7, IL-8 and Granulocyte Macrophage-Colony Stimulating Factor (GM-CSF), and 3 anti-inflammatory cytokines-(IL-4, IL-13 and IL-10). Briefly, stored plasma samples were thawed on ice and a two-fold serial dilution was prepared in 96 well plates using the assay diluent. Assay standards and reagents, after reconstitution and preparation, respectively, were added to the samples in volumes specified by the manufacturer’s protocol. After periods of washing and incubation, the plates were read using the Luminex^®^ 200^™^ system, running on the Xponent 3.1 software. The levels of the various cytokines were reported as either concentrations or median fluorescence intensity (MFI) for analytes with levels below the detection limit.

### Statistical analysis

All data analyses were performed using the GraphPad Prism software version 9.2.0. The Kruskal Wallis test was used to compare the differences in the plasma levels of the various cytokines in all patient categories for non-uniform distributed data. This was represented by graphs which also gave information on the degree of significance of the differences. Additionally, the receiver operating characteristic (ROC) curve analysis was performed to determine if any of the cytokines measured could discriminate between severe and uncomplicated malaria, giving information on their area under the curve (AUC) values as well as their specificities and sensitivities and cut-off values. For all analyses, they were considered significant if the p-value was less than 0.05.

## Results

### Demographic characteristic of participants

Samples from a total of 57 participants, consisting of 20 non-malarial febrile controls, and 37 children with malaria (27 with SM and 10 with UM) were used in this study. The participants consisted of 22 males and 35 females representing 38.60% and 61.40% respectively, which did not differ significantly (p = 0.37). There was no significant difference between the mean ages in the various groups (p = 0.32) (Table 1).

**Table 1:** Demographics of study participants

Characteristics	SM	UM	Non-malaria febrile control	P value
Sample size (n)	27 (47.37%)	10 (17.54%)	20 (35.09%)	NA
Sex (n)				
Male	8 (36.36%)	6 (27.27%)	8 (36.36%)	0.37
Female	19 (54.29%)	4 (11.43%)	12 (34.29%)	
Age (SD) years	1.5 (2.1)	4.6 (3.1)	3.7 (3.5)	0.32
Parasitaemia (IQR), /µl	54,683 (29,706–162,776)	36,228 (15,671–116,416)	NA	NA

*SM* severe malaria, *UM* uncomplicated malaria, *n* number of participants, *IQR* interquartile range, *SD* standard deviation, *NA* not applicable

### Pattern of pro-inflammatory responses in SM, UM and febrile control (FC) groups

**Fig. 1:**
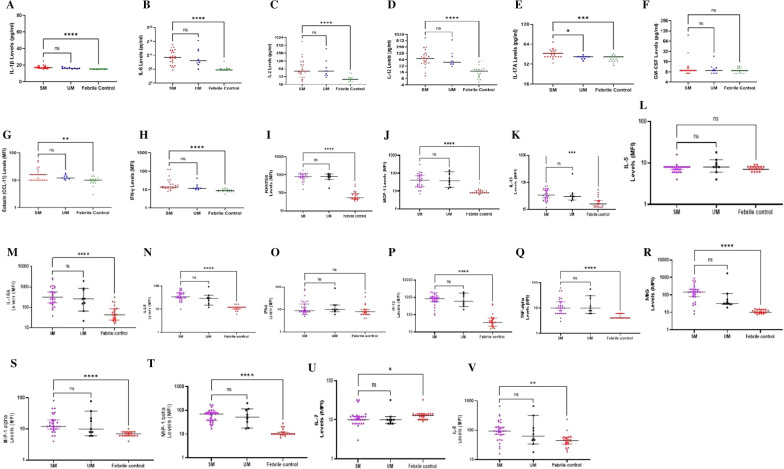
Profile of pro-inflammatory cytokines during the course of the various disease conditions. Scatter plot graphs are plotted showing the concentrations (pg/ml) of **A**–**F** IL-1β, IL-6, IL-2, IL-12, IL-17A, GM-CSF and median fluorescence intensities (MFI) of **G**–**V** Eotaxin, IFN-γ, RANTES, MCP-1, IL-15, IL-5, IL-1RA, IL-2R, IFN-α, IP-10, TNF, MIG, MIP-1α, MIP-1β, IL-7 and IL-8 in plasma samples collected from uninfected controls (n = 20), patients with severe malaria (n = 27) and uncomplicated malaria (n = 10). For each figure, the interquartile ranges (IQR) are shown as vertical bars and the median is shown as horizontal bars. Significant differences are denoted by *p < 0.05, **p < 0.01, ***p < 0.001, ****p < 0.0001, *ns* not significant

The first step for identifying cytokines for the discrimination of SM from UM cases was the measurement and comparison of their levels in all patient groups. As shown in Fig. 1, the circulating levels of the pro-inflammatory cytokines IL-1β, IL-6, IL-2, IL-12, Eotaxin/CCL-11, MCP-1, IL-15, IL-1RA, IL-2R, IP-10, TNF, MIG, MIP-1α, MIP-1β, IL-7, IL-8 and 1FN-γ differed significantly among the SM group compared to the FC group (p < 0.05). There was no significant difference in their levels among SM and UM groups except for IL-17A which was significantly higher (p = 0.0163) in the SM group compared to UM group. GM-CSF, IL-5 and IFN-α however showed no significant difference in their levels across the groups (p > 0.05).

### Pattern of anti-inflammatory responses in SM, UM and FC groups

**Fig. 2:**
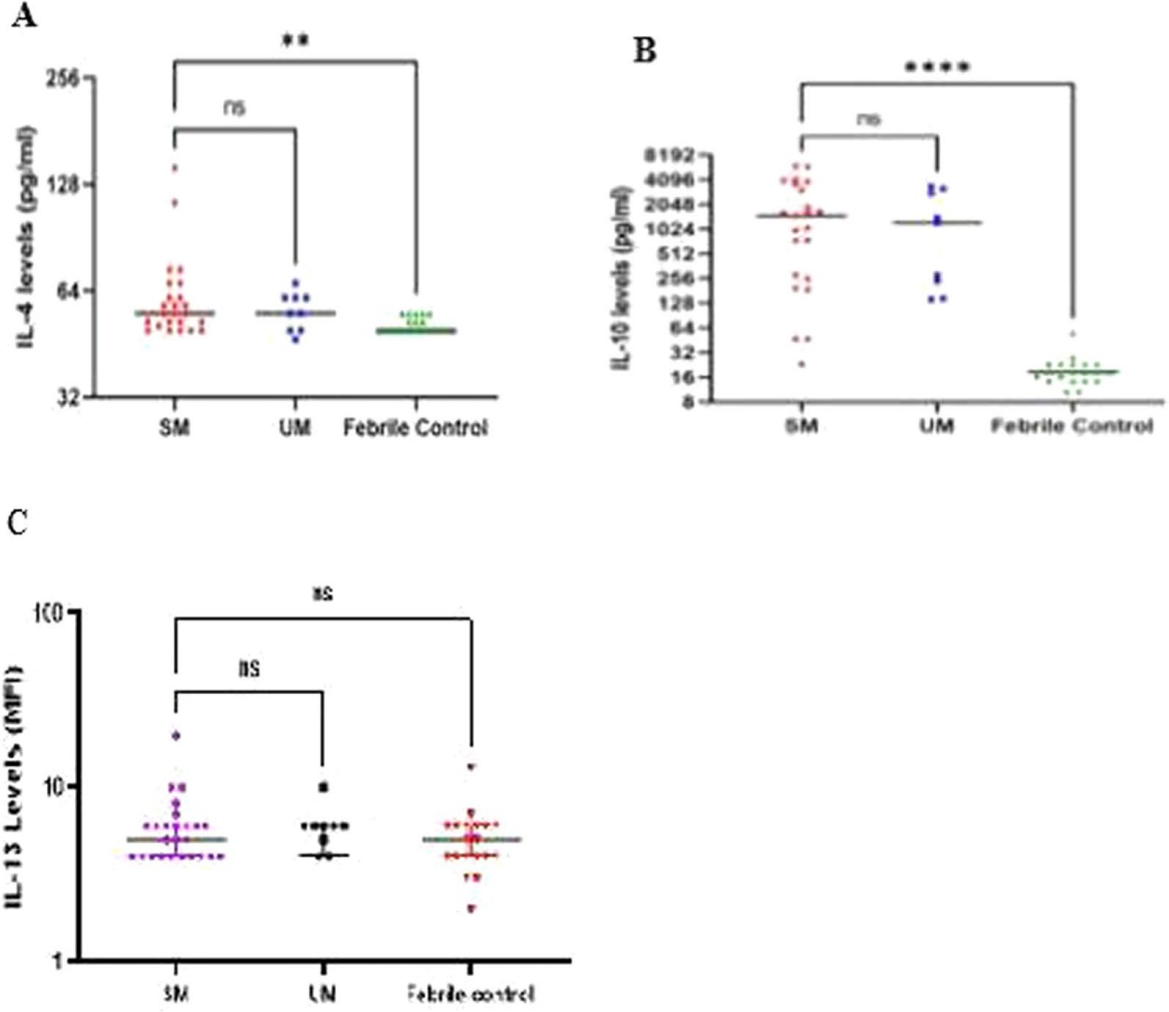
Profile of anti-inflammatory cytokines during the course of the various disease conditions. Scatter plot graphs are plotted showing the concentrations (pg/ml) of **A**–**B** IL-4, IL-10 and median fluorescence intensities (MFI) of **C** IL-13 in plasma samples collected from uninfected controls (n = 20), patients with severe malaria (n = 27) and uncomplicated malaria (n = 10). For each figure, the interquartile ranges (IQR) are shown as vertical bars and the median is shown as horizontal bars. Significant differences are denoted by *p < 0.05, **p < 0.01, ***p < 0.001, ****p < 0.0001, *ns* not significant

As shown in Fig. 2, the anti-inflammatory cytokines, IL-4 and IL-10, showed a significant difference in their levels among the SM group compared to the FC group (p = 0.0153 and p < 0.0001 respectively). There was however no statistically significant difference in their levels among the SM and UM groups. IL-13 however showed no significant difference in its levels across the groups (p ≥ 0.05).

### ROC curve analysis of the pro- and anti-inflammatory cytokines

**Fig. 3:**
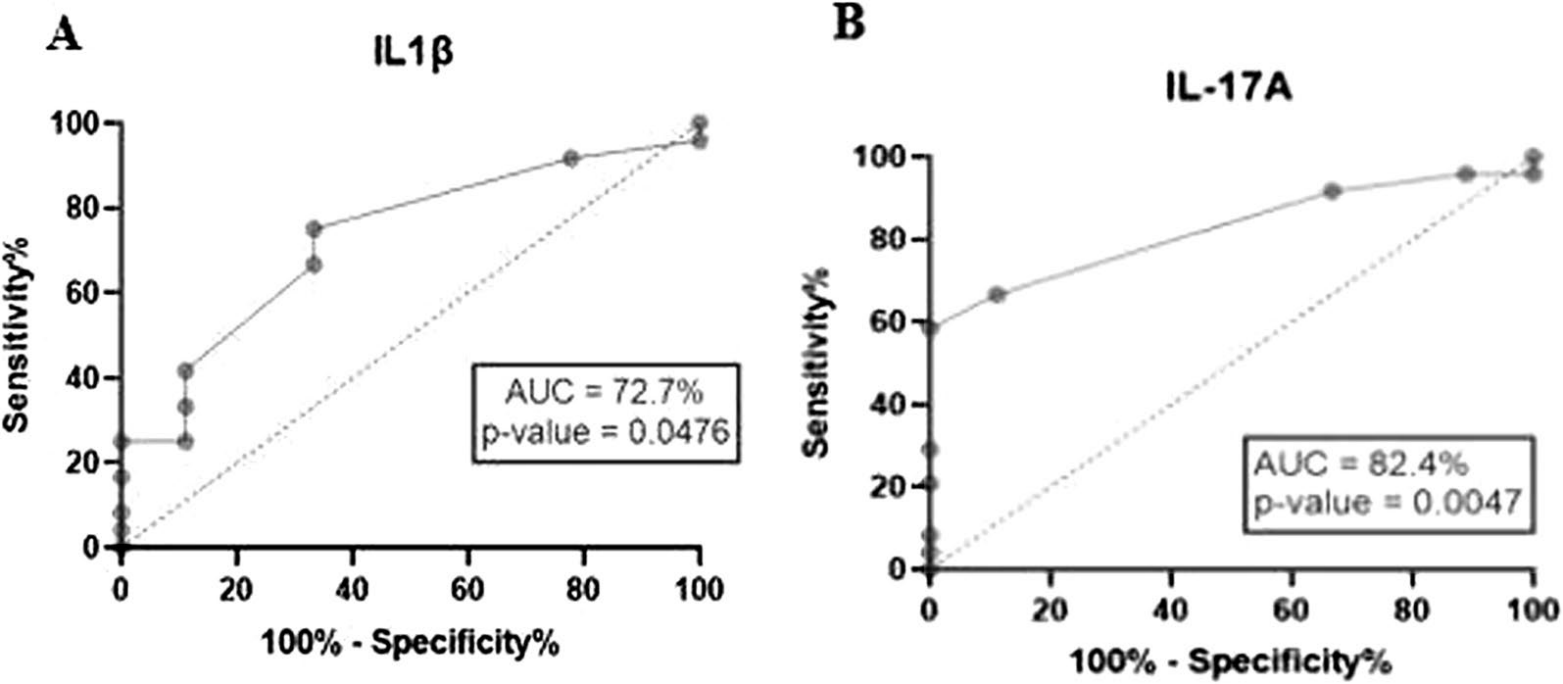
Graphs showing the ROC curves of **A**–**B** IL-1β and IL-17A

Using the SM group as patient sample and UM group as control, the ROC curve was used to quantify how accurately the pro-and anti-inflammatory cytokines can discriminate between the two patient states. The curve gave their area under the curve (AUC) values as well as their specificities and sensitivities and cut-off values (Table 2). Among all the cytokines measured, IL-17A and IL-1β showed significant AUC values (p = 0.0047 and p = 0.0476, respectively) and, therefore, have better potential to distinguish SM from UM cases (Fig. 3).

**Table 2:** ROC curve analysis of pro-and anti-inflammatory cytokines

Cytokines	Cut-off value (pg/ml)	AUC value (%)		P value	Sensitivity (%)	Specificity (%)	Likelihood ratio
IL-1β	** > 32.63**	**72.7**		**0.0476**	**75**	**66.67**	**2.25**
IL-6	> 795.4	54.2		0.6976	57.7	70	1.923
IL-2	> 110.4	53.2		0.7772	45.83	66.67	1.375
IL-12	> 100.2	59.7		0.3960	70.83	55.56	1.594
IL-17A	** > 85.34**	**82.4**		**0.0047**	**66.67**	**88.89**	**6.000**
GM-CSF	< 18.35	51.9		0.8598	73.08	50	0.462
IL-4	> 128.6	54.2		0.7160	25	88.89	2.250
IL-10	> 5880	57.2		0.5310	29.17	77.78	1.313

Cytokines	Cut-off value (MFI)	AUC value (%)	P value	Sensitivity (%)	Specificity (%)	Likelihood ratio
Eotaxin	> 15	56.7	0.5578	58.33	77.78	2.625
IFN-γ	> 12.50	68.1	0.1149	75	66.67	2.250
RANTES	> 169.5	51.9	0.8715	95.83	0	0.9583
IL-7	> 9.500	59.8	0.3593	72.41	40	1.207
MIP-1β	> 29.00	52.5	0.8265	85.19	22.22	1.095
MIP-1α	> 9.500	55.1	0.6479	77.8	44.44	1.4
MIG	> 26.50	71.8	0.0528	88.89	71.94	1
TNF	< 9.000	53.3	0.7701	37.04	55.56	0.8333
IP-10	> 229.0	51.0	0.5451	88.89	11.11	1
IL-2R	> 19.00	70.2	0.0734	96.3	33.33	1.444
IL-8	> 31.00	57.4	0.4892	89.66	10	0.9962
IL-5	< 5.000	66.7	0.1432	4	88.89	0.36
IL-15	> 14.50	53.3	0.6395	96	0	0.96
MCP-1	< 8.500	55.6	0.6256	4	100	NA
IL-13	< 4.500	56.4	0.5714	40	77.78	1.800
IL-1RA	> 85.50	56.9	0.5451	92	22.22	1.183
IFN-α	< 8.500	55.6	0.6237	46.15	77.78	2.077

*MFI* Median fluorescence intensity

## Discussion

Inflammatory cytokines have been implicated in the pathogenesis of several diseases despite their complex association with multiple regulatory pathways. Unregulated levels and a change in their expression patterns, is said to have potential as biomarkers of several diseases [9, 21]. Malaria is no different as cytokines have been reported to contribute to its progression from uncomplicated to severe [11]. Studies have identified cytokine profiles able to differentiate the various clinical manifestations of malaria (SM and UM), although their diagnostic potential in terms of cut-off values has not yet been established. In this study we aimed to corroborate detection of these immune markers among malaria groups and also explore their disgnostic potential in discriminating severe malaria from umcomplicated malaria and other febrile conditions.

We observed that the levels of the pro-inflammatory cytokines: IL-1β, IL-6, IL-2, IL-12, IL-17A, Eotaxin/CCL-11, 1FN-γ, RANTES, MCP-1, IL-15, IL-1RA, IL-2R, IP-10, TNF, MIG, MIP-1α, MIP-1β, IL-7, IL-8 and anti-inflammatory cytokines: IL-4 and IL-10 differed significantly among malaria-infected groups and the control groups. This is consistent with findings that inflammatory cytokines are elevated in peripheral blood during malaria infection [22]. There was no statistically significant difference in the levels of the pro-inflammatory cytokines among the SM and UM groups, except for IL-17A which was significantly higher in the SM group compared to the UM group. IL-17A is secreted by immune cells and is responsible for the mediation of granulopoiesis, infiltration of neutrophils and recruitment of T lymphocytes into peripheral tissues during inflammation [23]. High levels of IL-17A have been implicated in severe malaria anemia, multiple organ dysfunction, including acute renal failure during severe malaria [23]. In other infections such as COVID-19, increased levels of IL-17A, among other pro-inflammatory cytokines, have been associated with very poor prognosis including multiple organ dysfunction and death [24]. Mbengue et al. [25] reported that there is a strong induction in pro-inflammatory cytokine production during severe malaria as a result of accumulation of high concentrations of toxic parasite components at sites of *P. falciparum* infected red blood cells sequestration. In agreement, we observed that even though there was no significant difference in the pro-inflammatory levels between the SM and UM, the levels in the SM group seem to increase as that of the UM group decrease especially with IL-1β. These results are in line with several other observations that pro-inflammatory cytokines promote inflammation and tissue damage, which may imply that their levels correlate with malaria disease severity and death [11, 14, 26].

GM-CSF, IL-5 and IFN-α, though pro-inflammatory cytokines, did not show any significant difference in their levels in the SM group compared to the UM and FC groups. This discrepancy may be explained by the fact that GM-CSF only stimulates the synthesis and release of TNF during malaria but its concentration remains the same either at the time of diagnosis or after treatment of malaria [27].

Anti-inflammatory cytokines are produced to prevent the damaging effects of pro-inflammatory cytokines [15], therefore, an imbalance in the levels of pro- and anti-inflammatory cytokines contribute significantly to the pathogenesis of severe malaria [16]. Progression of uncomplicated malaria to severe is hence warranted by lower levels of anti-inflammatory cytokines compared to pro-inflammatory cytokines. This plausibly explains why there was no significant difference in the levels of IL-10, IL-13 and IL-4 among the SM and UM groups whereas the pro-inflammatory cytokine, IL-17A, was found to be increased in the SM group.

It is of great significance that biomarkers of malaria disease severity be identified to inform critical clinical management decisions including whether a patient has, or is progressing to severe malaria and therefore needs to be admitted, or referred to a tertiary healthcare facility for aggressive case management [28]. The diagnostic abilities of the cytokines to differentiate SM from UM was determined using the ROC curve analysis which quantifies how accurately medical diagnostic tests can discriminate between two patient states: diseased and non-diseased (controls) [29]. The area under the curve (AUC) is an effective measure of sensitivity and specificity that describes the inherent validity of diagnostic tests (in this case cytokines). AUC takes on values from 0 to 1 (0–100%), where a value of 0 indicates the perfect inability of a cytokine to differentiate disease from control group and a value of 1 (100%) indicates a perfectly accurate test, meaning the cytokine is perfect in the differentiation of diseased patients from control group [30]. An AUC of 0.5 (50%) suggests no discriminative ability of the cytokine, 0.7–0.8 (70–80%) is considered moderate, 0.8–0.9 (80–90%) is considered excellent, and more than 0.9 (90%) is considered perfect. Using the SM group as the disease state and the UM group as the control, the results obtained showed that IL-1β and IL-17A (AUC = 72.7% and 82.4% respectively) are good biomarkers for the discrimination of SM from UM cases. The AUC values of the other cytokines fell within the 0.5–0.6 (50–60%) range and therefore, were considered unable to distinguish SM from UM patients.

A major limitation of the study was low sample size, which was mainly due to the COVID-19 pandemic, which restricted the rate of patients’ visits to the hospital. Also, is this study was in partial fulfilment of an undergraduate degree in Medical Laboratory Sciences in the University of Ghana, there were time limitations for data collection.

## Conclusion

Increase in the levels of pro-inflammatory cytokines in the SM infected group compared to the UM and febrile control groups corresponds to increase in malaria disease severity. IL-17A is a good candidate biomarker for development of diagnostic tests for SM. IL-1β was also found to have good diagnostic potential and should be considered. This study augments the currently insufficient data on appropriate biomarkers for SM diagnosis.

## Data Availability

The raw datasets generated and/or analysed for this study will be made available by the authors on reasonable request.

## Abbreviations

AUC: Area under the curve
COVID-19: Coronavirus disease- 2019
FC: Febrile control
GM-CSF: Granulocyte macrophage-colony stimulating factor
HIV: Human immunodeficiency virus
IFN: Interferon
IL: Interleukin
IP: Interferon gamma-induced protein
IQR: Interquartile range
MCP-1: Monocyte chemoattractant protein-1
MFI: Median fluorescent intensity
MIG: Monokine induced by gamma interferon
MIP: Macrophage inflammatory protein
PML: Princess Marie Louise
RANTES: Unregulated upon activation, normal T-cell expressed and secreted
ROC: Receiver operating characteristic
SD: Standard deviation
SM: Severe malaria
TNF: Tumor necrosis factor
TGF: Transforming growth factor
UM: Uncomplicated malaria
